# Quantitative tandem mass-spectrometry of skin tissue reveals putative psoriatic arthritis biomarkers

**DOI:** 10.1186/1559-0275-12-1

**Published:** 2015-01-13

**Authors:** Daniela Cretu, Kun Liang, Punit Saraon, Ihor Batruch, Eleftherios P Diamandis, Vinod Chandran

**Affiliations:** Department of Laboratory Medicine and Pathobiology, University of Toronto, Toronto, Ontario Canada; Department of Pathology and Laboratory Medicine, Mount Sinai Hospital, Toronto, Ontario Canada; Department of Statistics and Actuarial Science, University of Waterloo, Waterloo, Ontario Canada; Department of Clinical Biochemistry, University Health Network, Toronto, Ontario Canada; Psoriatic Arthritis Program, Centre for Prognosis Studies in the Rheumatic Diseases, Toronto Western Hospital, 399 Bathurst Street, Room 1E 416, Toronto, Ontario M5T 2S8 Canada; Division of Rheumatology, Department of Medicine, University of Toronto, Toronto, Ontario Canada

**Keywords:** Psoriatic arthritis, Cutaneous psoriasis, Proteomics, Mass spectrometry, Biomarker

## Abstract

**Background:**

Psoriatic arthritis (PsA) is a distinct inflammatory arthritis occurring in 30% of psoriasis patients. There is a high prevalence of undiagnosed PsA in psoriasis patients; therefore, identifying soluble biomarkers for PsA could help in screening psoriasis patients for appropriate referral to a rheumatologist. Potential PsA biomarkers likely originate in sites of inflammation, such as the skin, and subsequently enter systemic circulation. Our goal was to identify candidate PsA biomarkers by comparing the proteome of skin biopsies obtained from patients with PsA to that from patients with psoriasis without PsA.

**Methods:**

Skin biopsies were obtained from involved and uninvolved skin of 10 PsA and 10 age/gender-matched psoriasis patients without PsA (PsC). Using strong cation exchange chromatography, followed by label-free quantitative tandem mass spectrometry, we characterized the proteomes of pooled skin samples. Extracted ion current intensities were used to calculate protein abundance ratios, and these were utilized to identify differentially regulated proteins.

**Results:**

Forty-seven proteins were elevated in PsA-derived skin compared to PsC-derived skin. Selected reaction monitoring assays were developed to quantify these potential PsA markers in individual skin samples, and 8 markers were confirmed in an independent sample set. ITGB5 and POSTN were measured in serum samples from 33 PsA and 15 PsC patients, using enzyme-linked immunosorbent assays. ITGB5 was significantly elevated in PsA serum (P < 0.01), and POSTN showed a trend. ITGB5 and POSTN correlated significantly in both patient groups (r = 0.472, P < 0.001).

**Conclusion:**

Proteomic analysis of PsA and PsC skin identified eight new candidate biomarkers. These markers need to be validated with a larger and independent cohort, in order to delineate their clinical utility in PsA patients. These proteins may also uncover unknown aspects of PsA pathobiology.

**Electronic supplementary material:**

The online version of this article (doi:10.1186/1559-0275-12-1) contains supplementary material, which is available to authorized users.

## Background

Psoriatic arthritis (PsA) is a distinct inflammatory arthritis, which takes its name from its association with the cutaneous, autoimmune inflammatory disease, psoriasis. It occurs in 30% of psoriasis patients and has a predicted prevalence of up to 1% in the general population. PsA is a complex, potentially disabling musculoskeletal disorder often arising early in age. Patients with PsA have an increased risk for a spectrum of co-morbidities, such as obesity, metabolic syndrome, diabetes and cardiovascular disease [1–3]. The diagnosis of PsA presents a challenge, largely due to its heterogeneous clinical presentation [4, 5]; however, early diagnosis and prognosis of PsA is essential for prevention of joint damage and disability [6].

The key to early diagnosis is a better recognition of PsA in patients with psoriasis, since its presence indicates a high risk for current or future development of PsA [3]. Soluble biomarkers represent an ideal means for screening patients for PsA. With improvements in high-throughput genomic platforms, a number of putative markers, ranging from susceptibility genes to mRNA profiles have been proposed [7–11]; however there exists no single or panel of specific markers, or mediating factor(s). Much of the research, therefore, focuses on the discovery and validation of PsA biomarkers [7, 12–14]. Identifying these factors would not only facilitate diagnosis and prognosis of PsA, but also provide further insight into disease pathogenesis.

Mass spectrometry (MS)-based quantitative proteomic approaches are well-suited for the discovery of protein mediators and biomarkers of disease [15, 16]. Specifically, label-free quantification (LFQ) methods have been recently optimized, where quantification is based on the differential peak intensity [extracted ion current (XIC)] of the peptides in each MS scan [17, 18]. Such experiments often result in tens to hundreds of candidate biomarkers, therefore, in this context of biomarker discovery, high-throughput mass spectrometry-based proteomics is a powerful tool for obtaining disease-specific proteome profiles of biological materials [19]. While human plasma represents a diverse proteome and is an excellent source of potential disease markers, proteins secreted by tissues are diluted in blood, and are often undetectable by current MS methods [19]. Much interest has been given to the analysis of proximal fluids and tissues, such as synovial fluid and skin [19–21].

Cutaneous psoriasis develops simultaneously or precedes the onset of PsA in up to 90% of PsA patients, and in order to move forward in the search for PsA screening biomarkers, we must consider the preceding cutaneous psoriasis stage in PsA patients. Therefore, hypothesizing that there are differences in the skin proteome of patients with PsA compared to those with psoriasis but without PsA (PsC), comparative analysis of skin between these two patient groups represents a reasonable experimental workflow. In a pilot study, our group demonstrated that proteins elevated in the inflamed skin, are likewise upregulated at the serum level, and may serve as putative markers of psoriasis [22].

In the current study, we performed label-free quantitation of skin proteins from PsA and PsC patients. Using selected reaction monitoring assays (SRM) we confirmed the elevation of some proteins in an independent set of samples from patients with PsA. Following a small-scale validation in serum using enzyme-linked immunosorbent assays (ELISA), we confirmed a significant elevation of β_5_ Integrin (ITGB5) in the serum of PsA patients. Periostin (POSTN) also showed a similar trend. These proteins may serve as potential PsA biomarkers and also shed new light into the pathogenesis of PsA.

## Results

### Delineating the PsA skin proteome

Our LC-MS/MS analysis yielded 1922 quantifiable proteins (Additional file 1: Table S1). Student’s t-tests, and a false discovery rate (FDR) threshold of 0.2, were utilized to identify upregulated proteins between the PsA and PsC lesional groups (PsA L, and PsC L, respectively). This comparative analysis generated a total of 62 proteins (Additional file 2: Table S2). Generally, these proteins exhibited a fold change ratio above 4.0. Comparison between PsA and PsC non-lesional skin (PsA N, and PsC N, respectively), at the same FDR, demonstrated elevated expression of 131 proteins (Additional file 2: Table S2). By comparing the proteins identified from the two independent analyses (PsA L vs PsC L, and PsA N vs. PsC N), only 7 of these proteins were common (Additional file 2: Table S2). These were PSME3, C1QC, RENBP, GRHPR, POLE, POSTN, and IGLV3-21.

Since our scope was to identify potential biomarkers present in PsA skin, we decided to focus on the 62 proteins overexpressed in the lesional PsA skin, which included the seven proteins elevated in non-lesional skin, since they are more likely to represent mediators and potential biomarkers of PsA. We further focused on proteins displaying strong expression in skin, bone, and immune regulatory cells, and excluded immunoglobulins, unknown proteins and high abundance serum proteins from further analysis. Additional file 3: Table S3 presents the final list of 47 proteins, corresponding fold changes (FC) between PsA and PsC samples, P-Values and FDR values. These were then assessed and quantified, using a multiplexed selected reaction monitoring assay, in individual skin samples included in the discovery set (Set I), as well as in an independent set of 10 samples (Set II).

### SRM Verification of putative markers in individual skin samples

We developed a multiplexed SRM assay to verify the differences in protein expression between lesional PsA and PsC skin. Assays were developed for 47 peptides representative of the 47 proteins with increased expression in the skin of PsA patients, as well as 4 peptides representing 2 housekeeping proteins (ACTB, TUBB) (Additional file 4: Table S4). Consistent with our LC-MS/MS analysis of the pooled samples, overexpression of 12 out of the 47 proteins was also verified in the individual PsA lesional skin samples (Set I), when compared to lesional PsC samples. Each sample consisted of two technical replicates. The mean fold change of each confirmed protein in the lesional and non-lesional PsA and PsC skin samples, as well as the associated P-values are depicted in Table 1. The proteins included C16ORF62, SNCA, LZIC, SRP14, ITGB5, POSTN, SRPX, FHL1, PPP2R4, CPN2, GPS1, and PAFAH1B2. The distribution of these, and the housekeeping proteins across the Set I skin samples, are represented in Additional file 5: Figure S1.

**Table 1:** Fold change (FC) of candidate markers in Set I and Set II* skin following SRM quantification

	***Set I***			***Set II***		
	Lesional		Non-lesional		Lesional	
Protein name	PsA:PsC FC ^**^	P-value ^***^	PsA:PsC FC	P-value	PsA:PsC FC	P-value
CPN2	17.4	<0.001	2.4	0.030	1.9	0.032
GPS1	6.0	0.014	1.2	0.385	17.9	0.008
C16ORF62	6.0	<0.001	5.3	0.007	3.9	0.667
FHL1	4.6	<0.001	3.1	0.021	2.2	0.016
SRPX	3.8	0.043	3.3	0.014	5.0	0.008
SNCA	3.6	<0.001	1.7	0.089	3.8	0.095
POSTN	3.5	0.001	2.8	0.013	7.5	0.032
PAFAH1B2	3.3	0.004	2.0	0.104	0.8	0.667
SRP14	3.0	0.019	1.3	0.570	2.4	0.016
ITGB5	2.7	0.006	3.5	0.017	4.2	0.032
PPP2R4	2.2	0.043	2.0	0.678	3.9	0.008
LZIC	2.0	0.036	1.6	0.212	1.2	0.413

*The description of Set I and Set II are given in the experimental methods section.

**Fold change (FC) represents the ratio of means of 10 (Set I) or 5 (Set II) skin samples per group. Data are based on normalized XIC ratios, as described in the experimental methods section. PsA, psoriatic arthritis; PsC, cutaneous psoriasis.

***P-Values were calculated using the non-parametric Mann-Whitney U test.

Additionally, we further confirmed the elevation of 8 of the aforementioned 12 proteins, in an independent set of 5 PsA, and 5 PsC lesional skin samples (Set II). In this case, we utilized a heavy-labeled peptide in order to obtain the absolute concentration of these peptides in the skin. The proteins included SRP14, ITGB5, POSTN, SRPX, FHL1, PPP2R4, CPN2, and GPS1. The mean fold changes and P-values corresponding to each protein from set II are also depicted in Table 1. Additional file 6: Figure S2 shows the distribution of the 8 proteins across the Set II skin samples, as well as of the housekeeping proteins. Since only 8 proteins were confirmed in the independent sample set, we decided to consider for further analyses only these 8 potential markers.

### Small-scale ELISA validation in serum

**Figure 1:**
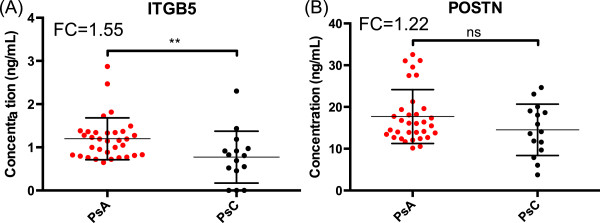
**Distribution of markers across the PsA and PsC serum sets.** Small-scale validation of ITGB5 **(A)** and POSTN **(B)** in PsA and PsC serum by ELISA. Dots represent serum samples from individual subjects; thin horizontal lines depict the mean, and vertical lines the standard deviation; FC represents the ratio of the mean concentration values corresponding to 33 PsA and 15 PsC serum samples. ** indicates P < 0.01; ns: non-significant. PsA, Psoriatic Arthritis; PsC, Cutaneous Psoriasis.

To validate the possible expression of markers in serum of PsA patients, we measured the levels of ITGB5 and POSTN in the serum of 15 PsC and 33 PsA patients. As shown in Figure 1, ITGB5 was significantly elevated in PsA, when compared to PsC patients (1.19 ± 0.5 compared to 0.77 ± 0.6; p < 0.01). The concentration of POSTN was not significantly different between the two groups (p > 0.05), but the levels in PsA serum showed an increasing trend, when compared to PsC serum (17.71 ± 6.4 compared to 14.53 ± 6.2). The mean FC and distribution of POSTN and ITGB5 in the PsA and PsC serum, are represented in Figure 1. We were unable to measure SRP14, SRPX, FHL1, PPP2R4, CPN2, and GPS1, due to unavailability of ELISA kits, antibodies, or protein standards.

### Correlation amongst biomarkers

**Figure 2:**
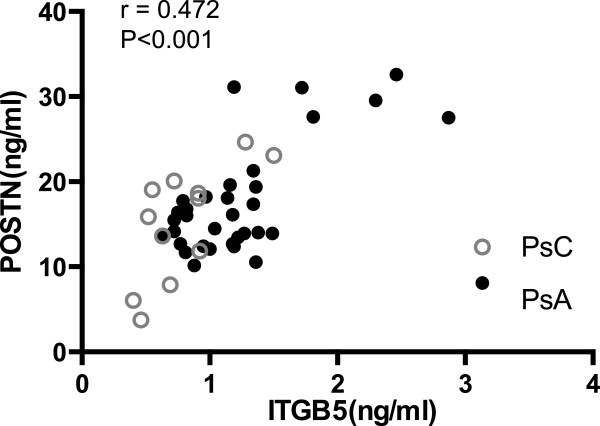
**Correlation between ITGB5 and POSTN across the PsA and PsC serum sets.** Dots represent serum samples from individual subjects. r indicates the Spearman’s rank correlation coefficient.

Spearman’s rank correlation coefficient was used to assess the correlation amongst markers for the PsA, and PsC serum groups. ITGB5 correlated significantly with POSTN in PsC serum (Spearman r = 0.637, P = 0.013), and in PsA serum (Spearman r = 0.433, P = 0.012). Figure 2 displays the correlation between POSTN and ITGB5 in all samples (Spearman r = 0.472, P < 0.001).

## Discussion

Recent advances in mass spectrometry-based proteomics facilitated the identification of putative novel biomarkers and mediators from a variety of tissues, and for various clinical applications [15, 16, 23]. In this study, we conducted high-throughput quantitative proteomics to identify differentially expressed proteins between skin derived from PsA and PsC patients. According to our filtering criteria, forty-seven proteins were elevated in the PsA group, when compared to the PsC group. Eight proteins were verified using a multiplexed selected reaction monitoring assay, in an independent skin sample set. None of these potential markers has been described before in the context of PsA or psoriasis. Since serum measurement of these proteins using SRMs is not efficient due to the high complexity of the fluid [18], we utilized available enzyme-linked immunosorbent assays to measure two biomarker candidates in serum (ITGB5, and POSTN).

Cumulative evidence strongly supports the involvement of Interleukin (IL)-23/IL-17 axis in the pathogenesis of PsA, and a number of compounds that target components of these pathways have been recently used in PsA clinical trials. IL-23 acts synergistically with IL-6 and TGF-β to promote rapid Th17 development and IL-17 release [24, 25], which, in turn, plays a central role in sustaining chronic inflammation [24]. Both POSTN and ITGB5 have been implicated with this pathway.

Periostin is a matricellular protein belonging to the fascilin family [26]. It interacts with several integrin molecules on cell surfaces, one of which is α_5_β_5_ Integrin, and provides signals for tissue development and remodeling [27]. More specifically, it is thought that POSTN interacts with α_5_β_5_ to induce pro-inflammatory cytokine production via the Akt/NF-κB signaling pathway [28]. In support of this, deficiency of POSTN or inhibition of α_5_ integrin (ITGA5) prevented development or progression of skin inflammation in an atopic dermatitis mouse model [28]. Additionally, POSTN has been shown to also act on immune cells, leading to their enhanced transmigration, chemotaxis, and adhesion [29], all of which further implicate this molecule in inflammatory processes relevant to PsA.

Apart from acting as a POSTN ligand (along with ITGA5), ITGB5 has also been implicated in rheumatoid arthritis, where it serves as a ligand for Cyr61 [30]. Cyr61 is a molecule secreted by fibroblast-like synoviocytes in the joint, and stimulates IL-6 production via ITGA5-ITGB5/Akt/NF-κB signaling pathway [29]. As described earlier, IL-6, along with IL-23 and TGB-β, trigger Th17 differentiation and IL-17 production [24, 25] in inflammatory processes, including PsA. Based on this data, we suggest that POSTN and ITGB5 are attractive molecules to investigate further as PsA biomarkers.

Following a small-scale serum validation of POSTN and ITGB5, we determined that only ITGB5 was significantly elevated in PsA serum, although POSTN also showed a trend. Additionally, Spearman correlation showed that serum ITGB5 correlates well with POSTN (r = 0.472, p < 0.001), which indicates that these two markers may, in the future, be used as part of a panel of markers to screen for PsA in PsC patients.

Although our findings are promising, the work reported here has some limitations. First, pooling of the skin samples in the discovery phase has advantages and disadvantages. While pooling allows for a more extensive coverage of disease heterogeneity, by increasing the likelihood of identifying proteins that are otherwise undetectable in some individual samples, it may also mask meaningful discrepancies among the different individual skin proteomes [19, 31]. To minimize this effect, samples were pooled to obtain two distinct pools in both, PsA and PsC groups. Additionally, all pool-derived candidates were further examined in all individual samples using a targeted and more sensitive mass spectrometry technique. As our results indicate, the correlation between the discovery data and SRM data is not ideal, whereby only 12 proteins were verified in the discovery set. This is due in part, to the “pooling” effects previously discussed. Our SRM data from individual samples identified several skin samples that had prominently higher protein concentrations. These samples most probably affected (increased) the final concentration of proteins in the pooled samples, which were used for our initial selection of candidates. Thus, analysis of individual samples via SRM assay allowed for elimination of such artifacts.

Second, the identified markers were only tested on a small number of serum samples (n = 48), and only 20 of these were distinct from the Set I and Set II sample sets. Therefore, to assess their accuracy in serum, these markers still need to be tested in a large-scale verification study. In addition, as discussed previously, only two of the existing eight proposed markers have been investigated in serum due to the lack of ELISAs or antibodies for the rest. Hence, strategies to enable measurement of the remainder of the markers will be employed in the future.

Finally, the 47 proteins we investigated using SRM, represent few of the candidates we have presently identified. Proteins that were elevated in non-lesional skin also represent interesting candidates, and must be measured in the future.

Taken together, our current observations are consistent with the notion that label-free quantitative LC-MS/MS can be utilized to quantify proteins in tissues, which can then be shed into the circulation and serve as serum markers of PsA. Further studies to validate these findings are underway, in a larger and independent serum cohort. Investigating these proposed markers further, may not only result in the identification of PsA screening biomarkers, but may also uncover aspects of PsA pathobiology that are currently unknown.

## Methods

### Skin proteomic analysis

#### Clinical samples

Samples were collected with informed consent after research ethics board approval from the University Health Network, Toronto, Canada. For the discovery phase, skin biopsies were obtained from 10 cases with PsA (6 males, 4 females; age range 38-73 years) and 10 PsC patients (6 males, 4 females; age range 28-77 years) (Set I). PsA patients had psoriasis and satisfied the CASPAR classification criteria [32], while the PsC patients were assessed by a rheumatologist, to exclude PsA. Patients were not undergoing treatment with methotrexate (MTX) or anti-TNF agents. One 6mm punch biopsy was obtained from unaffected (non-lesional) skin, and one from affected (lesional) skin from each PsA and PsC patient, amounting to a total of 40 samples.

For the verification (quantification) phase, an independent set of skin samples was obtained from 5 PsA patients (all males; age range 49-63 years), and 5 PsC patients (3 males, 2 females; age range 49-67 years) (Set II). Inclusion criteria were the same as described above.

For the small-scale validation, serum samples were obtained from 33 PsA patients (22 males, 11 females; age range 21-76 years), and 15 PsC controls (9 males, 6 females, age range 28-77 years). Inclusion criteria were the same as described above.

#### Pre-analytical sample processing

Skin samples were snap-frozen in liquid nitrogen and stored at -80°C until use. Samples were suspended in 0.05% RapiGest (Waters, Milford, MA, USA) buffer, and the tissue was homogenized and sonicated for protein extraction. The tissue lysates were spun at 11,000g and total protein was measured by the Bradford assay (Pierce Biotechnology, Rockford, IL, USA), in the resulting supernatants. Equal protein amounts from each sample were pooled to create eight different pools of five samples each [2 PsA lesional (PsA L) vs. 2 PsA non-lesional (PsA N) vs. 2 PsC lesional (PsC L) vs. 2 PsC non-lesional (PsC N)]. Proteins in each pool were denatured and reduced with 5 mM dithiothreitol at 60°C for 45 minutes and alkylated with 15 mM iodoacetamide, in the dark, at room temperature, for 45 minutes. Sequencing grade trypsin (Promega, Madison, WI, USA) was added in a 1:50 (trypsin: protein) ratio, and allowed to digest for 18 hours at 37°C. Trifluoroacetic acid (1%) was added to cleave RapiGest and inhibit trypsin activity, and samples were centrifuged at 11,000g for 20 minutes prior to high performance liquid chromatography (HPLC) using strong cation exchange (SCX), to reduce peptide mixture complexity. During the initial stages of SCX, buffer blanks were run after each sample, and the resulting fractions were assessed for peptide carryover; apart from peptides corresponding to a few high abundance proteins, we found very few peptides (data not shown). The peptide fractions resulting from HPLC-SCX were collected, pooled, and subjected to liquid chromatography and tandem mass spectrometry (LC-MS/MS), and was followed by protein identification and quantification. Experimental details regarding HPLC-SCX, LC-MS/MS, and protein identification and quantification, were previously published [31], and are also outlined in Additional file 7.

#### Bioinformatic analysis

To detect upregulated proteins in the PsA group, one-sided Student’s t-tests were used between PsA L and PsC L, and PsA N and PsC N after log transformation of XIC values. Only proteins displaying significant differences [False discovery rate (FDR) <0.2] were used for further analysis [33]. The FDR threshold was chosen such that if we repeat the procedure many times, 20% of the significant differences are expected to be false, on average. Averages of the replicate XIC values were calculated for each PsA L, PsA N, PsC L, and PsC N, and ratios (fold change: FC) of PsA L/PsC L, and PsA N/PsC N were computed.

Gene names of the upregulated proteins were checked against gene [BioGPS (http://biogps.org/#goto=welcome) [34]], and protein [Human Protein Atlas (http://www.proteinatlas.org/) [35] databases, to identify proteins with strong expression in PsA-associated tissues and cell types (skin, bone, immune cells). The Plasma Proteome Database (http://www.plasmaproteomedatabase.org/) [36] was employed to identify proteins present in high-abundance in serum, which could represent potential contaminants. Selected reaction monitoring (SRM) assays were developed for the top upregulated proteins in the lesional PsA group and housekeeping (ACTB, TUBB) proteins, and relative protein quantification was performed in individual skin samples to confirm their elevation in PsA skin.

### Verification of identified proteins using SRM

SRM methods were developed for verification of protein ratios in skin, following our previously published protocols [23, 31]. Details regarding the SRM assay development, skin sample preparation, and subsequent protein quantification are also outlined in Additional file 7.

### Validation of verified proteins

#### Enzyme-linked immunosorbent assays (ELISA)

The concentration of ITGB5 in serum was measured using an in-house developed protocol, the details of which are outlined in Additional file 7. Serum was diluted 10-fold.

The concentration of POSTN in serum was measured using a multiplexed Luminex Screening Assay (R&D Systems), according to manufacturer’s instructions. Serum was diluted 100-fold.

#### Statistical analysis

The Student’s t-tests were performed to analyze the LC-MS/MS data using R statistical software (http://www.r-project.org/). Other statistical analyses were performed using Graph Pad Prism v.6.0 for Mac (GraphPad Software, CA, USA). Results were analyzed using the nonparametric Mann-Whitney U test. P-values less than 0.05 were considered statistically significant. Spearman's rank correlation coefficient was used to assess the correlations between serum levels of POSTN, and ITGB5. Concentrations are reported in the text as mean ± standard deviation (SD).

**Figure 3:**
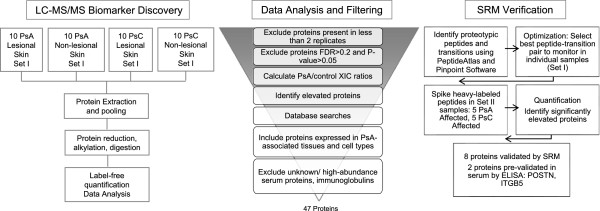
**Summary of the experimental design.** See text and abbreviations for more details.

The overall experimental design is provided in Figure 3.

## Electronic supplementary material

Additional file 1: Table S1: RAW data listing 1922 proteins identified by LC-MS/MS, in lesional and non-lesional PsA and PsC skin. (XLSX 450 KB)

Additional file 2: Table S2: Unfiltered list of elevated proteins in lesional and non-lesional PsA skin, as identified by LC-MS/MS. The red font represents data and calculations ycorresponding to PsA lesional skin, while the blue font indicates the PsA non-lesional skin data points and calculations. Proteins commonly elevated in the lesional and non-lesional PsA groups are depicted in the last column. (XLSX 44 KB)

Additional file 3: Table S3: Fold change (FC) of 47 elevated proteins in PsA skin compared to PsC skin, as identified by LC-MS/MS. ^*^FC represents the ratio of the mean XIC values of 10 skin samples per group (PsA L vs. PsC L); ^**^N/A indicates that a ratio could not be compiled since the protein was absent in PsC skin; ^***^P-Values were calculated using the student's t-tests; ^****^FDR represents the false discovery rate of each protein. (XLSX 11 KB)

Additional file 4: Table S4: List of 47 filtered and 2 housekeeping proteins, and the corresponding peptide sequences and transitions that were monitored in the multiplexed SRM assay. The sequence and transitions of the spiked-in heavy peptide are also depicted in the last three rows. (XLSX 13 KB)

Additional file 5: Figure S1: Distribution of markers across the PsA and PsC skin Set I. Dots represent skin samples from individual subjects; thin horizontal lines depict the mean, and vertical lines the SD. **** indicates P < 0.0001; ***P < 0.001; **P < 0.01; *P < 0.05; ns:non-significant. (PDF 122 KB)

Additional file 6: Figure S2: Distribution of markers across the PsA and PsC skin Set II. Dots represent skin samples from individual subjects; thin horizontal lines depict the mean, and vertical lines the SD. **** indicates P < 0.0001; ***P < 0.001; **P < 0.01; *P < 0.05; ns:non-significant. (PDF 70 KB)

Additional file 7: **Supplementary Materials and Methods.** (DOCX 26 KB)

## Abbreviations

ACE: Angiotensin-converting enzyme
ACN: Acetonitrile
ACTB: Beta-actin
ACTH: Adrenocorticotropic hormone
C16ORF62: Chromosome 16 open reading frame 62
C1QC: Complement C1q
CASPAR: Classification criteria for Psoriatic Arthritis
CPN2: Carboxypeptidase N, polypeptide 2
ELISA: Enzyme-linked immunosorbent assay
FA: Formic Acid
FC: Fold change
FDR: False discovery rate
FHL1: Four and a half LIM domain1 1
GPS1: G-protein pathway suppressor 1
GRHPR: Glyoxylate reductase/hydroxypyruvate reductase
HPLC: High-performance liquid chromatography
IGLV3-21: Immunoglobulin Lambda Variable 3-21
IL: Interleukin
ITGA5: Integrin alpha 5
ITGB5: Integrin-beta 5
LC-MS/MS: Liquid chromatography and tandem mass spectrometry
LFQ: Label-free quantification
LZIC: Leucine Zipper and CTNNBIP1 Domain-Containing Protein
MS: Mass-Spectrometry
MTX: Methotrexate
PAFAH1B2: Platelet-activating factor acetylhydrolase 1b, catalytic subunit 2
POLE: DNA Polymerase II Subunit A
POSTN: Periostin
PPP2R4: Protein phosphatase 2A activator, regulatory subunit 4
PsA L: Lesional Psoriatic Arthritis
PsA N: Non-lesional Psoriatic Arthritis
PsA: Psoriatic Arthritis
PsC L: Lesional Cutaneous Psoriasis
PsC N: Non-lesional Cutaneous Psoriasis
PsC: Cutaneous Psoriasis
PSME3: Activator of Multicatalytic Protease Subunit 3
SCX: Strong cation exchange
SD: Standard deviation
SNCA: Alpha synuclein
SRM: Selected reaction monitoring
SRP14: Signal recognition particle 14kDa
SRPX: Sushi-repeat containing protein
TFA: Trifluoroacetic acid
TGF: Transforming growth factor
TNF: Tumor necrosis factor
TUBB: Beta-tubulin
XIC: Extracted Ion Current.
