# Supplementary figures and images for: Quantitative tandem mass-spectrometry of skin tissue reveals putative psoriatic arthritis biomarkers
Source: Clin Proteomics. 2015 Jan 13;12(1):1. doi: 10.1186/1559-0275-12-1 (PMC4304122; doi:10.1186/1559-0275-12-1)

Normalized XIC

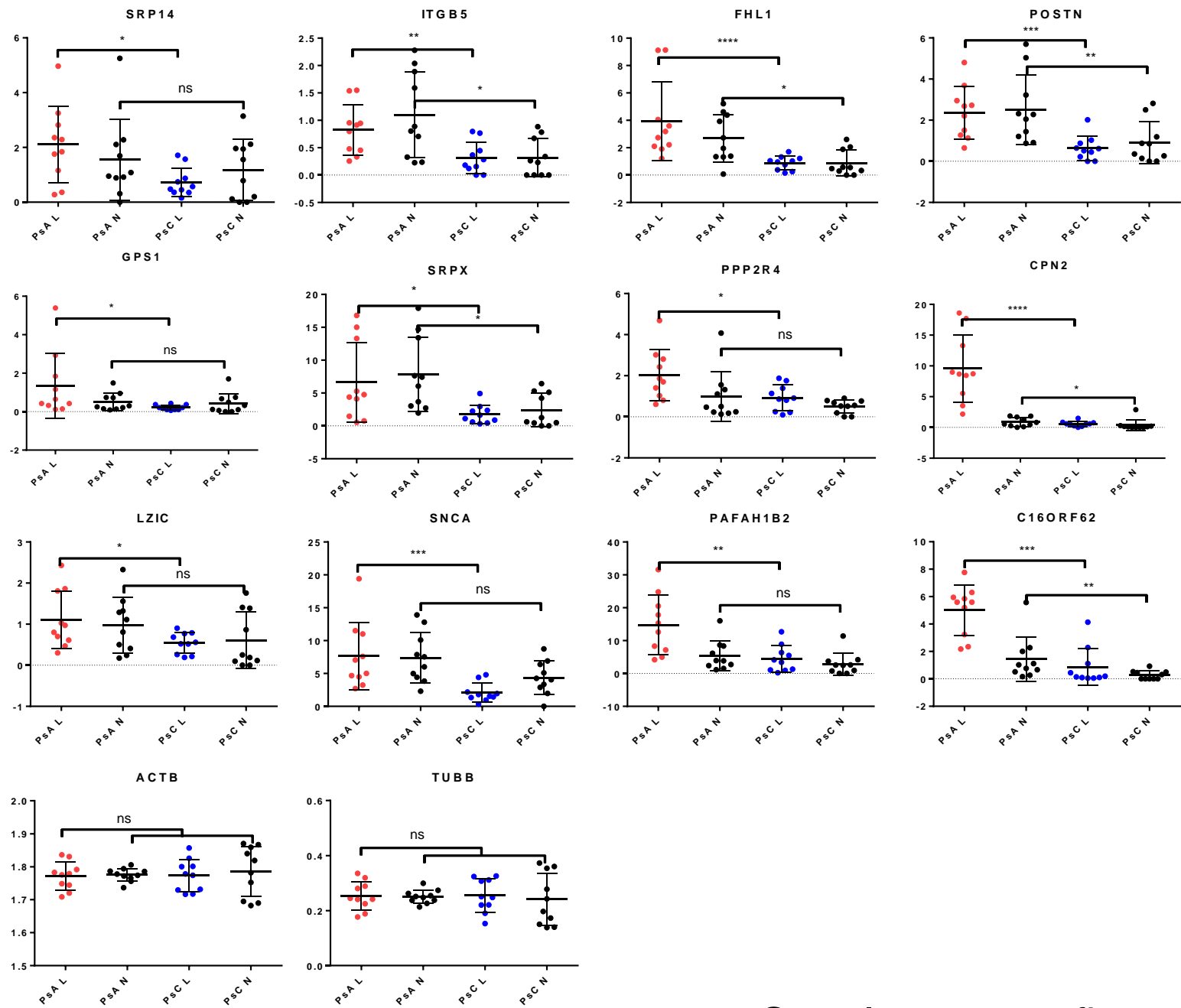

Supplementary figure 1.

Supplement: Supplementary file 5 — Additional file 5: Figure S1: Distribution of markers across the PsA and PsC skin Set I. Dots represent skin samples from individual subjects; thin horizontal lines depict the mean, and vertical lines the SD. **** indicates P < 0.0001; ***P < 0.001; **P < 0.01; *P < 0.05; ns:non-significant. (PDF 122 KB) [file 12014_2014_86_MOESM5_ESM.pdf]

Concentration

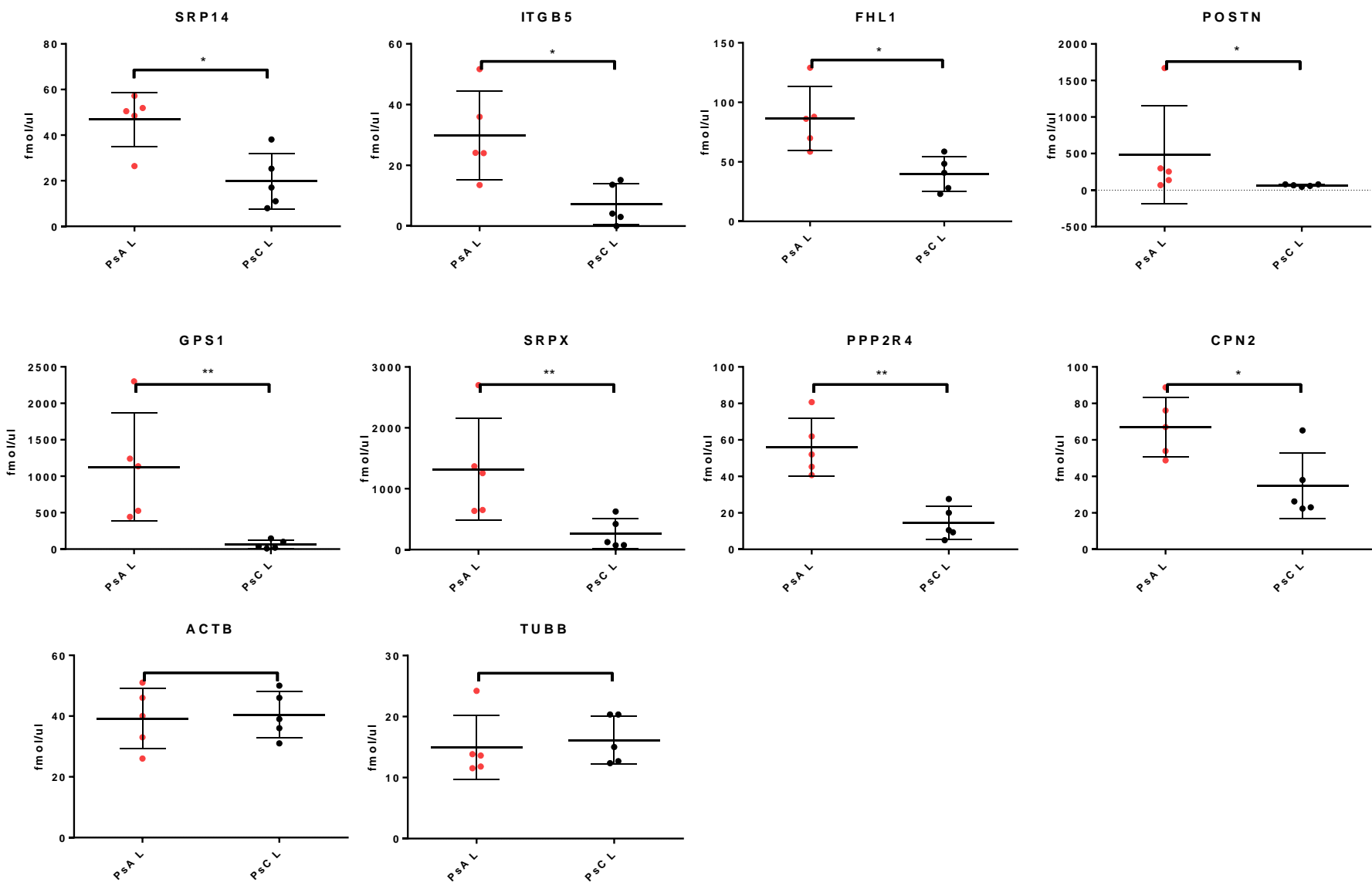

Supplementary figure 2.

Supplement: Supplementary file 6 — Additional file 6: Figure S2: Distribution of markers across the PsA and PsC skin Set II. Dots represent skin samples from individual subjects; thin horizontal lines depict the mean, and vertical lines the SD. **** indicates P < 0.0001; ***P < 0.001; **P < 0.01; *P < 0.05; ns:non-significant. (PDF 70 KB) [file 12014_2014_86_MOESM6_ESM.pdf]
