# Supplementary material for: Quantitative tandem mass-spectrometry of skin tissue reveals putative psoriatic arthritis biomarkers
Source: Clin Proteomics. 2015 Jan 13;12(1):1. doi: 10.1186/1559-0275-12-1 (PMC4304122; doi:10.1186/1559-0275-12-1)
Supplement: Supplementary file 7 — Additional file 7: Supplementary Materials and Methods. (DOCX 26 KB) [file 12014_2014_86_MOESM7_ESM.docx]

**SUPPLEMENTARY MATERIALS AND METHODS**

**Skin Proteomics Analysis**

*HPLC-SCX*

Digested samples were diluted 1:2 in mobile phase A SCX buffer [0.26 M formic acid (FA), 10% acetonitrile (ACN); pH 2.5] and loaded directly onto a 500 μL loop connected to a PolySULFOETHYL A column (2.1 mm inside diameter × 200 mm length; 5 μm particle size; 200 °A pore size; The Nest Group Inc., MA, USA), containing a silica-based hydrophilic, anionic polymer (poly-2-sulfotheyl aspartamide). An Agilent 1100 HPLC system was used for fractionation. A 60 minute gradient was employed with a linear gradient starting at 30 minutes and consisting of mobile phase A and mobile phase B (0.26 M FA, 10% ACN, 1 M ammonium formate; pH-4.5) for elution of peptides (flow rate 200uL/min). The fractionation was monitored at a wavelength of 280 nm and performed in duplicate. Fractions were collected every two minutes from 20 to 55 minutes, and those with a low peak absorbance were pooled, resulting in a total of 12 fractions per sample. This amounted to a total of 96 SCX fractions, which were then subjected to liquid chromatographic and tandem mass spectrometric analysis (LC-MS/MS). SCX column and system performance was ensured by running a quality control peptide mixture consisting of 1ug/uL Alpha Bag Cell peptide, 1ug/uL Fibrinogen fragment, 5ug/uL Human ACTH, and 5ug/uL ACE Inhibitor (American Protein Company, CA, USA) after every sample.

*LC-MS/MS*

The SCX fractions were purified through C-18 OMIX Pipette Tips (Agilent Technologies, Germany), to remove impurities and salts, and eluted in 5 μL of 65% MS buffer B [90% ACN, 0.1% FA, 10% water, 0.02% trifluoroacetc Acid (TFA)] and 35% MS buffer A (5% ACN, 0.1% FA, 95% water, 0.02% TFA). The samples were diluted to 85 μL in MS buffer A and injected into a nano-LC system (Proxeon Biosystems, FL, USA) connected online to an LTQ-Orbitrap (Thermo Fisher Scientific, USA). A 90 minute linear gradient reversed phase chromatography using MS buffer A and MS buffer B was performed at a flow rate of 400 nL/min to resolve peptides on a C-18 column (75um x 5 cm; Proxeon Biosystems, FL). The MS parameters were: 300 Da minimum mass, 4000 Da maximum mass, automatic precursor charge selection, 10 minimum peaks per MS/MS scan; and 1 minimum scan per group. The full MS scan was acquired in the Orbitrap, with subsequent MS2 scans on the top six parent ions in the linear ion trap in data-dependent mode. Dynamic exclusion, monoisotopic precursor selection and charge state screening were enabled such that only +2 and +3 ions were subjected to MS2 fragmentation. XCalibur software v.2.0.7 (Thermo Fisher Scientific, USA) was used for data acquisition.

*Protein Identification and Quantification*

Raw files corresponding to PsA L, PsA N, PsC L, and PsC N data sets were uploaded into MaxQuant v.1.2.2.2 (www.maxquant.org) [1] and searched with Andromeda (built into MaxQuant) [2] against the non-redundant IPI.Human v.3.71 database (86, 309 sequences; released March 2010). Search parameters included a fixed carbamidomethylation of cysteines and variable modifications of methionine oxidation and N-terminal acetylation. Data was initially searched against a “human first search” database with a parent tolerance of 20 ppm and a fragment tolerance of 0.5 Da in order to calculate and adjust the parent tolerance to 5 ppm for the search against the IPI.Human fasta file. During the search, the IPI.Human fasta database was randomized and the false detection rate was set to 1% at the peptide and protein levels. Data was analyzed using “Label-free quantification” checked, and the “Match between runs” interval was set to 2 min. Proteins were identified with a minimum of one unique peptide. “Unique and razor peptides” were included for quantification of proteins, the sum of which was represented in “LFQ Intensity” columns. Columns corresponding to the XIC value of each protein in replicate PsA L, PsA N, PsC L, and PsC N were averaged, and used to calculate PsA/PsC lesional and non-lesional ratios (fold change; FC). Statistical analysis of the dataset was performed using a Student’s t-test, as described in the main text.

**Verification of identified proteins using SRM**

*SRM Assay Development*

PeptideAtlas (http://www.peptideatlas.org/) was utilized to select 3–4 tryptic peptides for the proteins of interest. The uniqueness of peptides was verified by BLAST (https://blast.ncbi.nlm.nih.gov/Blast.cgi). To identify peptide fragments (transitions) to monitor, in silico peptide fragmentation was performed using Pinpoint software (Thermo Fisher Scientific, USA) and 5–6 transitions were selected for each peptide. For method optimization, digested pooled samples of PsA lesional skin, used in our LC-MS/MS analysis, were loaded onto a C-18 column (Proxeon Biosystems, FL, USA) coupled to a triple quadrupole mass spectrometer (TSQ Vantage; Thermo Fisher Scientific, USA), and approximately 2500 transitions were monitored in 25 subsequent runs. Three transitions were used for subsequent quantification. The identity of peptides was confirmed first, by prediction of retention times using SRRCalc 3.0, which predicts the retention time according to the hydrophobicity of each peptide. Second, identifying unique transitions using SRM Collider (http://www.srmcollider.org/srmcollider/srmcollider.py), and observing the co-elution of 3 of these transitions for each peptide analyzed [3].

*Skin sample preparation*

Fifty µg of total protein of each skin sample were denatured by heat, reduced, alkylated, and trypsin-digested as described previously. In sample set II, 500 fmol of a heavy-labeled peptide (Thermo Fisher), were also added as an internal standard. The heavy peptide sequence was LGPLVEQGR, where the C-terminal arginine was labeled with ^13^C and ^15^N. The resulting peptides were purified through C-18 OMIX Pipette Tips (Agilent), and eluted in 3µL of 65% MS buffer B and 35% MS buffer A. Samples were diluted to 40µL of MS buffer A, randomized, and loaded onto a C-18 column coupled to a triple quadrupole mass spectrometer. Details regarding SRM assays, and subsequent protein quantification are outlined in Supplementary Materials and Methods.

*SRM Assays*

SRM assays were developed on a triple-quadrupole mass spectrometer (TSQ Vantage) using a nanoelectrospray ionization source (nano-ESI, Proxeon Biosystems, FL), as previously described [4]. Briefly, a 60 minute, three-step gradient was used to load peptides onto the column via an EASY-nLC pump (Proxeon Biosystems, FL) and peptides were analyzed by a multiplexed SRM method. Quantification, in skin set I was performed after normalization against a set of 4 peptides corresponding to 2 housekeeping proteins, to offset technical variations. Each sample was analyzed in duplicate, using a 60-minute method, whereby 153 transitions were monitored. Quantification in SF set II was performed following normalization against the spiked-in heavy-labeled peptide, as described earlier. Each sample was analyzed in duplicate, using a 60-minute method, whereby 156 transitions were monitored. Reproducibility of the SRM signal was confirmed by running a quality control solution of 0.1 fmol/μL BSA after every 10 runs.

*SRM Protein Quantification*

Raw files recorded for each sample were analyzed using Pinpoint software (Thermo Fisher) [5], and peptide XIC were extracted. Pinpoint was used for identification and visualization of transitions. In the first skin set, in order to control for technical variation between the samples, XIC corresponding to each endogenous peptide replicate were divided by the XIC corresponding to the average of the two housekeeping peptides. This value was then averaged amongst the two replicate runs, to obtain “Normalized XIC”. In skin set II, in order to control for technical variation between the samples and obtain a more robust quantitative value for the proteins of interest, the XIC value corresponding to each endogenous peptide, was normalized to the XIC value corresponding to the spiked-in heavy peptide, in order to obtain a light:heavy (L:H) ratio. Since we added a known amount of heavy peptide to our samples prior to analysis, we used the L:H ratio to calculate the relative concentration of each endogenous peptide corresponding to the proteins of interest.

**Validation of Verified Proteins**

*ITGB5 ELISA*

Sheep anti-human ITGB5 polyclonal antibody (R&D Systems, Minneapolis MN, USA) was immobilized in a 96-well clear polystyrene plate by incubating 100 μL of 0.75 ng/μL capture antibody in PBS (137 mM NaCl, 2.7 mM KCl, 8.1 mM Na_2_HPO_4_, 1.5 mM KH_2_PO_4_, pH 7.4) overnight. The plates were washed three times with wash buffer (5 mmol/L Tris, 150 mmol/L NaCl, 0.05% Tween® 20, pH 7.8), after which the plate was blocked by adding 300 μL of 1% BSA in PBS to each well and incubated with shaking at room temperature for 60 min. The plates were then washed three times with wash buffer and incubated with 100 μL per well of ITGB5 recombinant protein standards (R&D Systems), or serum samples with shaking at room temperature for 2 h. ITGB5 standards and serum samples were diluted in 1% BSA in PBS, with all serum samples diluted 10-fold. After incubation, the plates were washed three times with wash buffer and incubated with 100 μL per well of biotinylated sheep anti-human ITGB5 detection antibody (R&D Systems) (0.25ng/μL detection antibody in 1% BSA in PBS) with shaking at room temperature for 2 h. After washing the plates three times with wash buffer, 100 μL of streptavidin-conjugated horseradish peroxidase solution (diluted 200-fold in 1% BSA in PBS) was added to each well and incubated for 15 min with shaking at room temperature. A final wash of three times with washing buffer was followed by the addition of 100 μL of 3,3′,5,5′-Tetramethylbenzidine (Sigma Aldrich, St, Louis, MO, USA) per well and incubated with shaking at room temperature for 10 min. The chromogenic reaction was stopped with the addition of 50 μL of 2 mol/L hydrochloric acid solution per well. Subsequently, the absorbance of each well was measured with the Wallac Envision 2103 Multilabel Reader (Perkin Elmer, MA) at 450 nm, standardized to background absorbance at 540 nm. Final serum concentrations were calculated by multiplying by the dilution factor.

**RELEVANT REFERENCES:**

1.Cox J, Mann M: **MaxQuant enables high peptide identification rates, individualized p.p.b.-range mass accuracies and proteome-wide protein quantification*.*** *Nat Biotechnol* 2008, **26**: 1367-1372.

2. Cox J, Neuhauser N, Michalski A, Scheltema RA, Olsen JV, Mann M: **Andromeda: a peptide search engine integrated into the MaxQuant environment.** *J Proteome Res* 2011, **10**: 1794-1805.

3.Röst H, Malmström L, Aebersold R. **A computational tool to detect and avoid redundancy in selected reaction monitoring.** Mol Cell Proteomics. 2012, **11**:540-549.

4. Martínez-Morillo E, Cho C-KJ, Drabovich AP, Shaw JL, Soosaipillai A, Diamandis EP: **Development of a multiplex selected reaction monitoring assay for quantification of biochemical markers of down syndrome in amniotic fluid samples.** *J Proteome Res* 2012, **11**: 3880-3887.

5. Campbell J, Rezai T, Prakash A, Krastins B, Dayon L, Ward M, Robinson S, Lopez M: **Evaluation of absolute peptide quantitation strategies using selected reaction monitoring.** *Proteomics* 2011, **11**: 1148-1152.
